# Herding conditions related to infectious keratoconjunctivitis in semi-domesticated reindeer: a questionnaire-based survey among reindeer herders

**DOI:** 10.1186/s13028-016-0203-x

**Published:** 2016-04-12

**Authors:** Morten Tryland, Solveig Marie Stubsjøen, Erik Ågren, Bernt Johansen, Camilla Kielland

**Affiliations:** 1 Arctic Infection Biology, Department of Arctic and Marine Biology, UiT-Arctic University of Norway, Stakkevollveien 23, 9010 Tromsø, Norway; 2 Department of Health Surveillance, Section for Disease Prevention and Animal Welfare, Norwegian Veterinary Institute, P.O. Box 750, Sentrum, 0106 Oslo, Norway; 3 Department of Pathology and Wildlife Diseases, National Veterinary Institute, 751 89 Uppsala, Sweden; 4 Northern Research Institute-Tromsø, Tromsø Science Park, 9294 Tromsø, Norway; 5 Department of Production Animal Clinical Sciences, Faculty of Veterinary Medicine and Biosciences, Norwegian University of Life Sciences, Ullevålsveien 72, 0454 Oslo, Norway

**Keywords:** Eye disease, IKC, Keratoconjunctivitis, Ocular disease, Reindeer, Risk factors, Traditional knowledge, Questionnaire

## Abstract

**Background:**

Infectious keratoconjunctivitis (IKC) in Eurasian semi-domesticated reindeer (*Rangifer tarandus tarandus*) is a multifactorial disease, associated to infectious agents such as Cervid herpesvirus 2 (CvHV2) and various species of bacteria, but environmental factors may also be necessary to initiate the disease. Little effort seems to have been invested in addressing the herder`s experience with this disease. An information letter with a link to an online questionnaire was sent to 410 herding community representatives in Norway and Sweden.

**Results:**

Sixty-three herders responded, 76 % of these having reindeer in Norway and 24 % in Sweden. Thirty-three herders (55 %) responded that they had seen this disease during the preceding year (2010) and 23 (38 %) that they had seen it in previous years (2009 or earlier). The majority (67 %) claimed that only 1–5 animals in their herd were affected at one time, whereas three herders (7 %) responded that more than 30 animals had been affected. No environmental factor could be singled out as significantly associated with the appearance of IKC, but when categorizing the number of contact herds for each herd (i.e. sharing pastures, corrals etc.), IKC was observed more often in herds with many (>25) contact herds. The questionnaire revealed that a veterinarian is not always available for reindeer herders, but also that a veterinarian seldom is contacted for this disease. None of the herders practiced isolation of a diseased animal from the rest of the herd when IKC was observed. Slaughter was the action most commonly initiated by the herders in response to IKC, whereas the veterinarian usually prescribed antibiotics, usually an ophthalmic ointment, alone or combined with systemic treatment. The herders claimed that IKC and other diseases had less importance than predators concerning loss of animals.

**Conclusions:**

IKC is to be considered a common disease, observed in 55 % of the herds (2010), typically affecting 1–5 animals, although larger outbreaks (>30 animals) occur. The herders usually slaughtered affected animals rather than consulting a veterinarian for medical treatment.

## Background

Infectious keratoconjunctivitis (IKC), known as infectious bovine keratoconjunctivitis in cattle (IBK), is a contagious eye disease and the most important eye disease in cattle worldwide [1]. The disease also occurs in other livestock [1, 2] and wildlife [3–5] and is generally regarded as a multifactorial disease. In cattle, the Gram negative bacterium *Moraxella bovis* is regarded as the main cause of the disease. Also, *Moraxella bovoculi* and a range of other bacteria, viruses, and environmental conditions seem to be involved [1, 6].

**Fig. 1:**
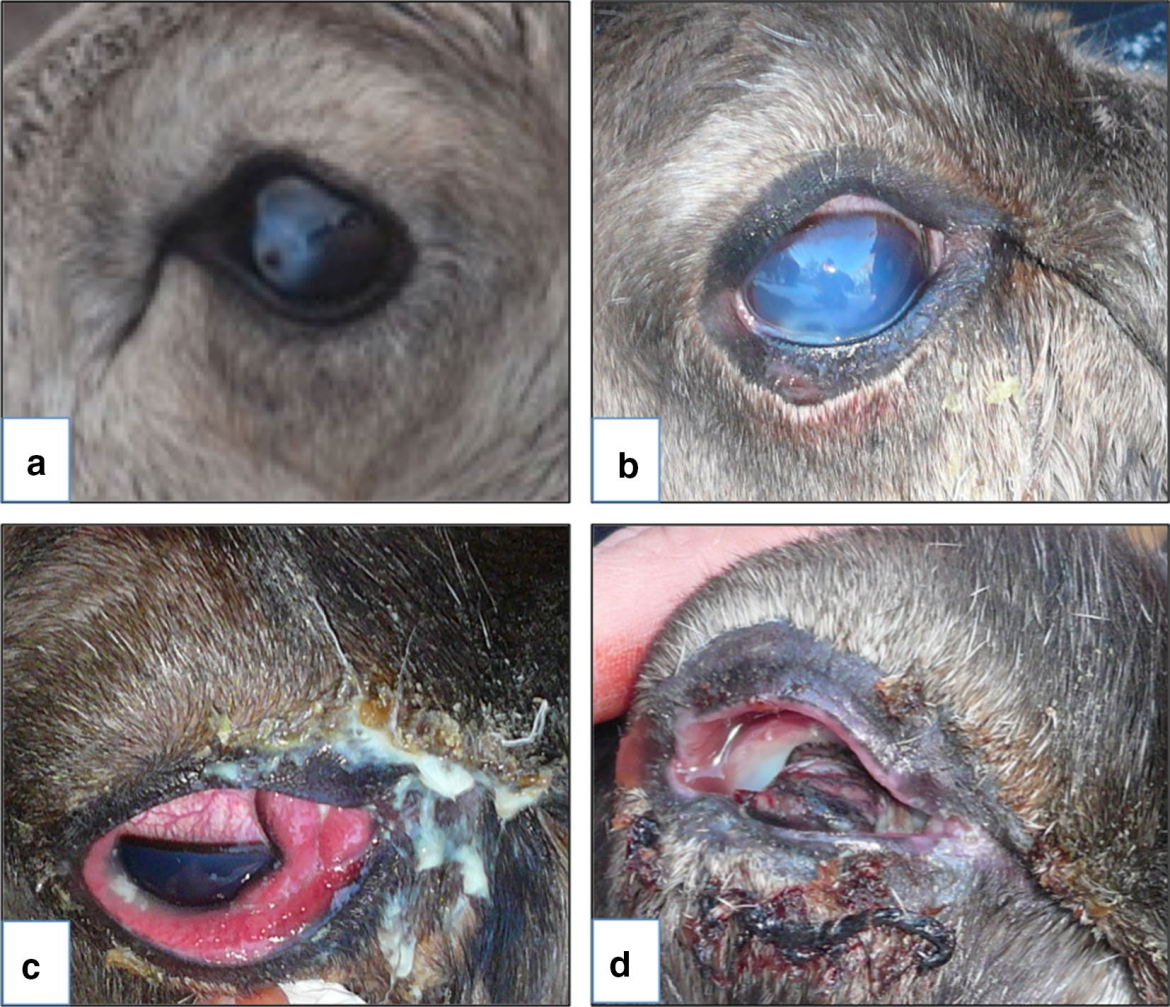
Clinical symptoms representing different stages of eye disease in reindeer, developing into infectious keratoconjunctivitis and a total destruction of the eye and permanent blindness (see text for further descriptions)

IKC has been reported in Eurasian semi-domesticated reindeer (*Rangifer tarandus tarandus*) in Norway, Sweden, and Finland since the late 19th century [7]. Increased lacrimation and discoloration of the fur below the affected eye(s) is an early sign of IKC in reindeer. Although neither keratitis nor conjunctivitis may be prominent at this stage, reindeer owners, especially those who have experienced outbreaks in their herd, often notice these initial symptoms. Conjunctivitis and keratitis is usually present, accompanied by corneal oedema, giving the eye a cloudy and bluish appearance. When the disease progresses, periorbital oedema becomes prominent, often followed by corneal ulcers, panophthalmitis and loss of the lens and other structures of the eye, leading to permanent blindness (Fig. 1). Animals may recover spontaneously from the early stages of the disease [8], but sometimes, in severe outbreaks affecting many animals in a herd, IKC cause mortalities with severe losses for the herders [9, 10].

In reindeer, a variety of bacteria, such as *Trueperella pyogenes*, *Staphylococcus* spp., *Escherichia coli*, *Moraxella ovis* and others have been isolated from reindeer with IKC, although most of the studies have addressed agents present at late clinical stages of disease [9, 11–15]. In early stages of IKC, we identified the reindeer alphaherpesvirus (Cervid herpesvirus 2; CvHV2) as the primary cause of an outbreak in 2009 [10], a virus that is considered enzootic in herds of semi-domesticated reindeer in Fennoscandia (for a review, see [16]). Thus, other factors than the presence of infectious agents may be decisive of whether the disease occurs or not.

Reindeer herders live very close to nature and are therefore to a great degree exposed to the potential effects of climate change [17]. With the documented changes of the global climate [18], it is expected that temperature and precipitation will increase in the arctic and sub-arctic regions, including the reindeer herding regions of Fennoscandia. An increase in rain-on-snow and freeze–thaw events during winter, may make the winter pastures less available for reindeer, being covered under heavy, hard-crusted snow or ice [17]. One way to mitigate challenging winter pasture conditions is to conduct supplementary feeding of the animals, either by bringing feed to the reindeer pastures, or by feeding the animals in corrals. Corralling and feeding animals will, however, expose the herd to stress which may affect their immunological competence and contribute to the animals being more susceptible to infections. At the same time, the transmission of infectious agents between animals are facilitated due to increased animal-to-animal contact and shared feed and water [15, 19, 20]. Previous outbreaks of IKC, both in Sweden [9] and Norway [10, 15] have indeed occurred in corrals during supplementary feeding. Climate change and mitigation by increased feeding may thus contribute to increased prevalence of diseases, such as IKC.

The aim of this questionnaire survey was to gather traditional knowledge and experience from reindeer herders in Norway and Sweden regarding IKC in reindeer, and identify herding conditions and management factors that might be associated to IKC.

## Methods

### Subjects

Registered reindeer herders (n = 410), were invited through their reindeer herding unit representatives (siida leaders; Norway, sameby chairperson; Sweden) to participate in this study. The questionnaire was made available to the participants by sending a letter that provided information on the survey and a web address with access to the online questionnaire.

### Questionnaire

A permission to conduct an anonymous questionnaire survey among Sami reindeer herders were obtained from Norwegian Social Science Data Services (NSD). The questionnaire was designed using the online program Questback^®^ and translated from Norwegian to Swedish and Sami (Northern Sami). Data from each of the responders were anonymously stored in a database on a web-server. After completing the survey, data were exported to Stata SE/111 for Windows (Stata Corp., College Station, TX, USA) where primary processing of data and quality check was conducted.

### Demographics and management

Demographic data collected from the reindeer owners were gender, age and years of experience as a reindeer herder. Herd data consisted of country (Norway or Sweden), herding district, herd size and number of contact herds, the latter defined as how many other herds the actual herd shared pastures, corrals, and transport vehicles with during the year. Herders were also asked about pasture conditions in 2010, which was the year before the survey was sent out, and to evaluate and grade the importance of weather conditions, such as precipitation (summer and winter) and winter temperatures. In addition, methods and time used for translocation of animals between seasonal pastures and the use of supplementary feeding were addressed. Additionally, owners were asked about important causes of loss of animals, other diseases than IKC, prophylactic anti-parasitic treatment, and their access to veterinary expertise.

### Appearance and severity of IKC

Specific questions about IKC were related to when the disease normally appeared (season). Pictures of eye infections and IKC in reindeer were provided (Fig. 1), grossly representing four categories of severity; A: a stage which can represent a trauma or a condition developing into IKC, with increased lacrimation and discoloration of the cornea, B: corneal oedema, with severely discoloration (bluish/whitish) of the cornea, increased lacrimation, moderate periorbital oedema C: progressed periorbital oedema and shedding of pus, and D: inflammation with perforation and destruction of the eye resulting in permanent blindness. Herders were asked if they had seen similar conditions, which stage of the disease they had seen the most, how often, and their estimation of how many animals that were affected at a time. They were also asked about how the disease was managed, if a veterinarian generally was available for the herding district, and the choice of treatment, initiated either by the herder or by the veterinarian.

### Statistical analysis

Primary data analysis was undertaken using Questback^®^. For further statistical and graphical analysis, data were transferred to Stata SE/111 for Windows (Stata Corp.). Demographic data was tabulated and percentages calculated. Simple Chi square test was used to look for associations between IKC and possible risk factors. In all analyses, statistical significance was considered with a *P* value <0.05.

## Results

### Demographic data of the questionnaire survey

**Fig. 2:**
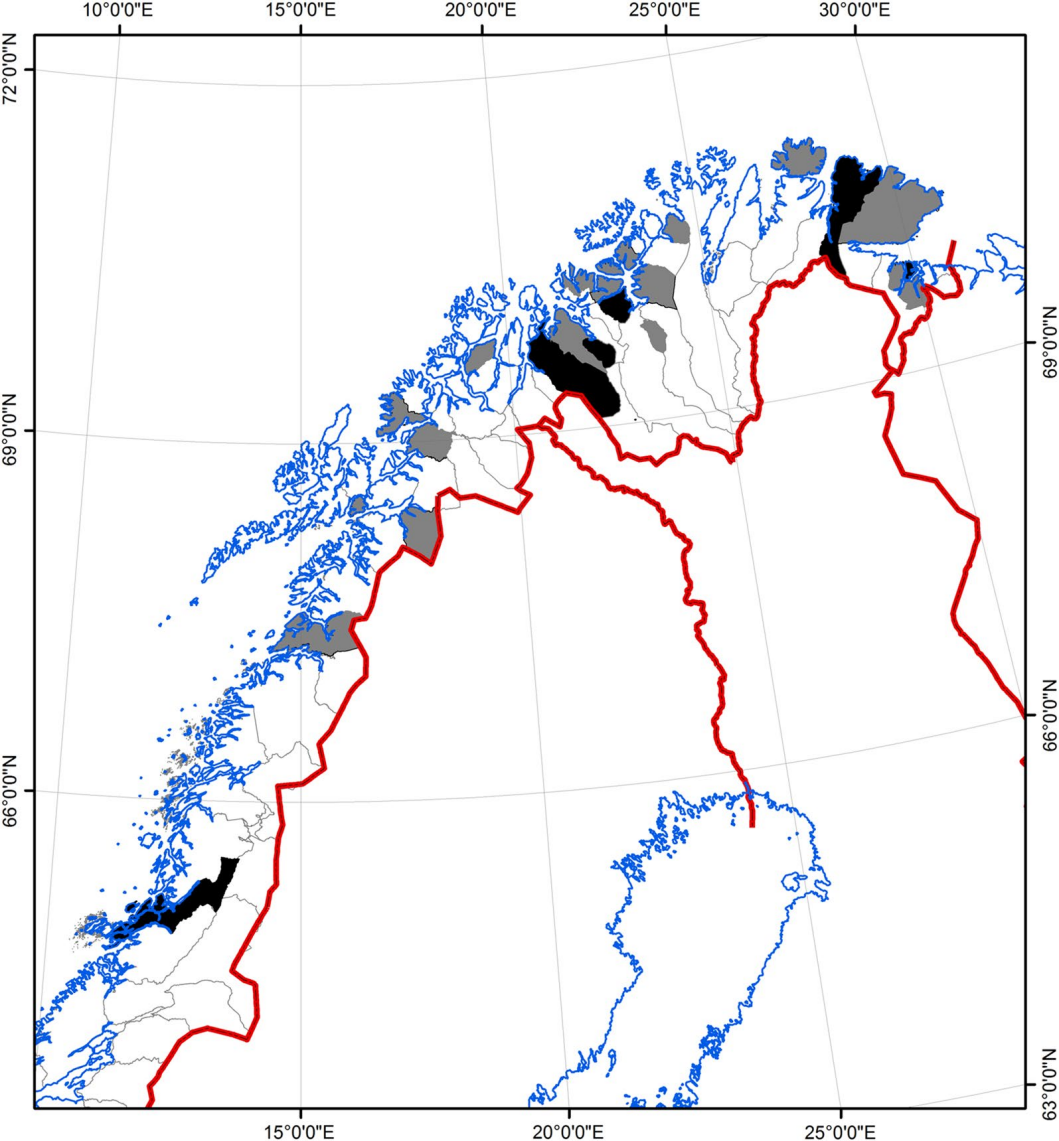
Map of Northern Norway, indicating the reindeer herding districts represented by reindeer herders responding to which district they represented (n = 37; no such information were given by the Swedish herders). *Grey* = 1 herder, *black* = 2 herders

From the 410 reindeer herders in Norway and Sweden that received the letter with the link to the questionnaire, 63 (16 %) responded (Table 1). The majority of the respondents (76 %) had reindeer in Norway, and only Norwegian herders informed about which reindeer-herding district they represented (Fig. 2). Most of the respondents were male herders aged between 31 and 50 years old, and 40 % of the respondents had a herd size between 251 and 500 animals. Almost 30 % of the herds were in contact with more than 25 other herds during a year of herding (summer and winter pastures, shared corrals and transports etc.).

**Table 1:** General demographic data of the questionnaire survey among Norwegian and Swedish reindeer herders regarding the disease infectious keratoconjunctivitis in reindeer

Parameter	Category	Number	Percentage^a^
Country (herders/animals)	Norway	48	76
	Sweden	15	24
Gender	Male	52	78
	Female	8	22
Age (years)	<30	4	6
	31–40	19	31
	41–50	19	31
	51–60	13	21
	>60	7	11
Experience as reindeer herder (years)	<10	9	15
	10–20	10	16
	21–30	16	26
	31–40	15	24
	>40	12	19
Herd size (approximate number of animals)	1–250	9	15
	251–500	23	38
	501–750	15	25
	751–1000	2	3
	1001–1250	6	10
	1251–1500	2	3
	>1500	4	7
Number of contact herds (shared pastures, corrals, transport etc.)	0	2	3
	1–5	14	23
	6–10	10	16
	11–15	8	13
	16–20	7	11
	21–25	3	5
	>25	18	29

^a^ Since decimals are omitted, the sum (percentage) is not always 100 for each question

### Appearance of IKC

Regarding the last time herders saw clinical symptoms resembling IKC (Fig. 1), 11 herders (18 %) answered that they had never observed this, 26 (43 %) answered that they had observed it during the previous year (2010) and 23 (38 %) said they had seen it 2 years ago or in previous years (but not in 2010) (Table 2).

**Table 2:** Answers about the appearance of infectious keratoconjunctivitis in reindeer based on 63 reindeer herder respondents, 48 in Norway and 14 in Sweden

Question	Category	Number	Percentage^a^
When did you observe eye disease as illustrated?^b^	Never observed	11	18
	Last year	26	43
	2 years ago	8	13
	3 years ago	5	8
	4 years ago or more	10	17
If this eye disease was observed last year (2010), how many animals were affected?	1–5	31	67
	6–10	7	15
	11–15	1	2
	16–20	3	7
	21–30	1	2
	>30	3	7
How does the disease normally look like in your herd?	A	23	47
	B	22	45
	C	1	2
	D	0	0
	All types occur	3	6

^a^ Since decimals are omitted, the sum (percentage) is not always 100 for each question

^b^ Refers to illustrations given in (Fig. 1a–d)

Most of the respondents that had seen IKC among their animals (49 %) claimed that September–November was the most common season of occurrence during the year, whereas 26 % answered June–August, 17 % September–February, and 9 % March–May.

**Fig. 3:**
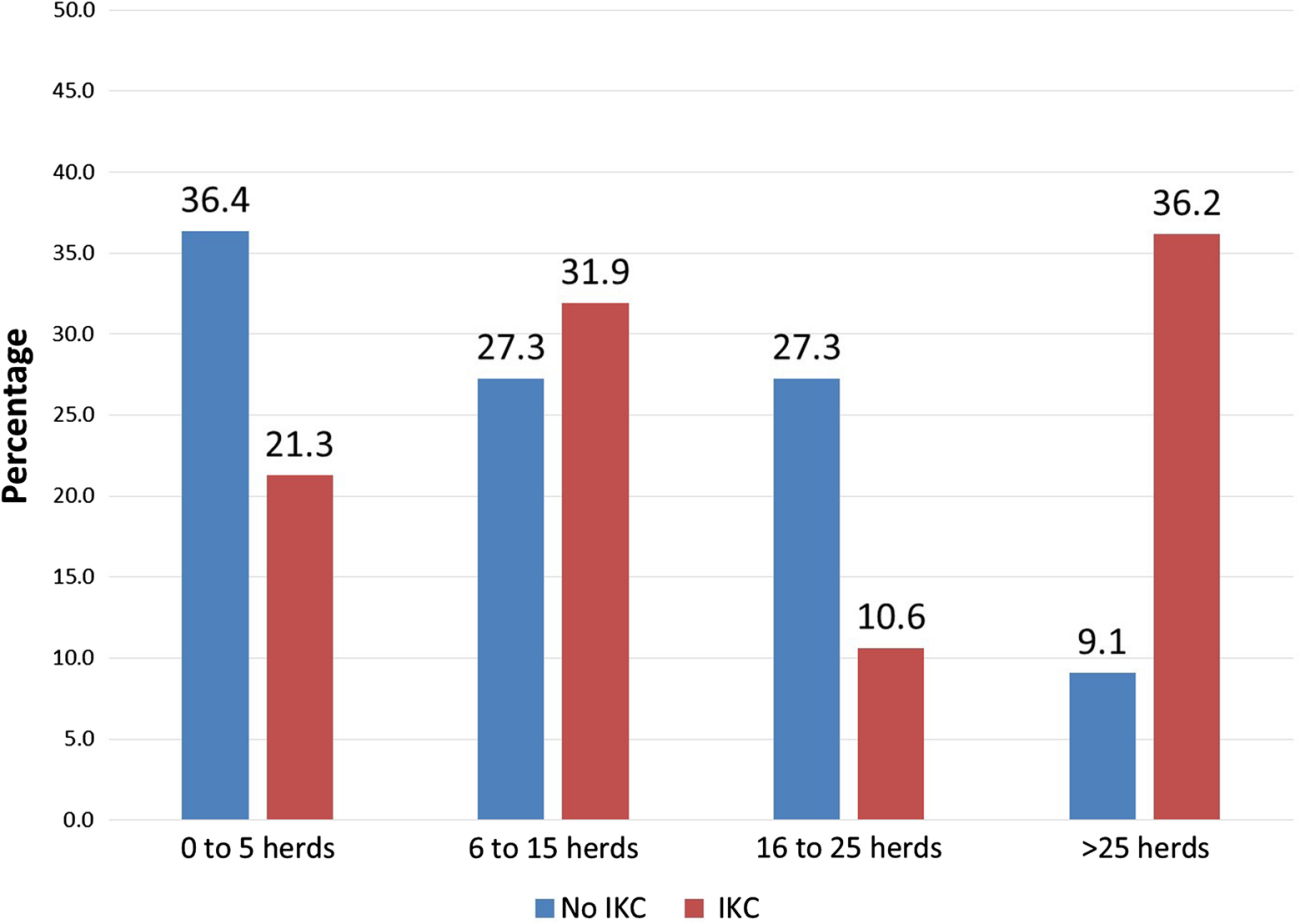
The association between the occurrence of infectious keratoconjunctivitis among reindeer in a herd and the number of contact herds for that particular herd, i.e. sharing pastures, corrals, transport vehicles etc. during the year

When IKC was observed in the herd, 31 herders (67 %) answered that typically 1–5 animals were affected, 7 (15 %) answered that 6–10 animals were usually affected, whereas 8 (17 %) had experienced that IKC could affect more than 10 animals in their herd. Only three herders reported that more than 30 animals had been affected by IKC at one time (Table 2). There was no significant association between the occurrence of IKC in a herd and the number of contact herds. However, when categorizing the number of contact herds into four groups, a visual trend appeared, since among the herds with >25 contact herds, 35 % reported that they had seen IKC, whereas only 10 % report that they had not seen IKC (Fig. 3).

Based on the responding herder`s experience from 2010, the severity of the IKC disease symptoms was categorized as mild, with 17 % as A and 45 % as B (Fig. 1). Only three had seen the category C, and none of the herders had seen category D, except for three herders that reported to have seen all categories. IKC in reindeer was mainly detected when animals were gathered, corralled and handled for identification, slaughter or other purposes (89 %).

Most of the reindeer herders responding to the survey (n = 35, 58 %) said they had access to a veterinarian, whereas 19 (31 %) said that they had access to a veterinarian some times, and 6 (10 %) claimed that they had no access to a veterinarian in their region. When animals were observed with IKC in a herd, a majority of the herders (56 %) chose slaughter instead of medical treatment (Table 3). The most common treatment against IKC in reindeer initiated by a veterinarian (according to the herders) was the use of ophthalmic ointment containing antibiotics (41 %). Other types of treatment are listed in Table 4.

**Table 3:** Management actions taken by the herder when the disease infectious keratoconjunctivitis occurred in semi-domesticated reindeer (*Rangifer t. tarandus*). *Question* what do you do when this disease occurs in a single/a few animals in the herd?

Management action taken by the herder	Percentage of responders (n = 57)
No measures taken	23
Separate the affected animal from the herd	0
Initiate a treatment (by the owner)	12
Slaughter/euthanize the animal	56
Consult a veterinarian	7
Other action (not specified)	2

**Table 4:** Management (by the veterinarian, according to the herder) of infectious keratoconjunctivitis in semi-domesticated reindeer (*Rangifer t. tarandus*). *Question* if a veterinarian is initiating measures—what measures are most common?

Measures by the veterinarian	Percentage of responders (n = 34)^a^
Ophthalmic ointment containing antibiotics	41
Systemic treatment with antibiotics	3
Both systemic and local (ointment) antibiotic treatment	6
Slaughter the animal (consumption)	56
Euthanasia	12
Other action (not specified)	38

^a^ Multiple answers were given by some responders

### Environmental conditions and pasture

**Fig. 4:**
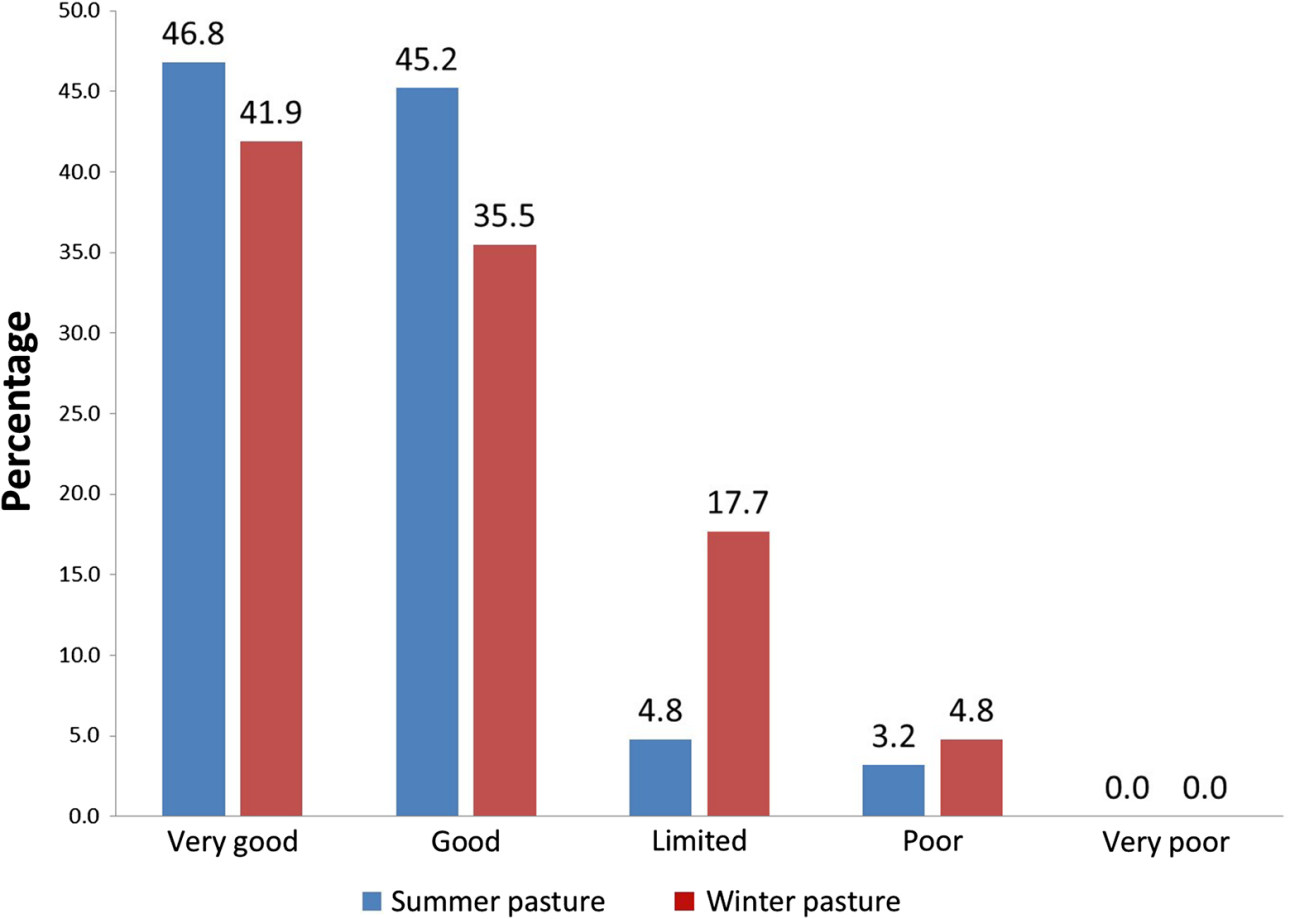
Most of the reindeer herders (percentages given) responding to the survey characterized their summer and winter pastures as good or very good in 2010, whereas 8 and 23 % of the herders evaluated their pastures as poor or limited during summer and winter, respectively

Of all the respondents, 55 % answered that weather and climatic conditions had a crucial impact on herding on a daily basis, whereas 42 % answered that weather and climate conditions sometimes were of importance. Only 3 % answered that such conditions were not important for their herding. The amount of precipitation as rain was regarded as important, both in the summer (37 %) and in winter (40 %). Regarding temperature fluctuations, 42 % of the respondents thought that large changes of temperature during winter have an impact on their herd, whereas wind, coldness, heat and dry periods were regarded as less important. A vast majority of the respondents characterized their summer and winter pastures as good or very good, for the year of 2010 (Fig. 4).

### Herding and feeding

Most owners (23 %) used 1–2 weeks to translocate their animals from summer to winter pasture, whereas 19 % said that they used 1–5 h, and 12 % that they used more than 6 weeks. Most animals were herded by motorized vehicles, such as quad bikes in the summer (51 %) and snowmobiles in the winter (88 %), but 25 % of the herders said they also herded their animals on foot. On winter pasture, 21 % fed their animals regularly, whereas 64 % fed them only occasionally. The most prevalent feeds used were dry hay (32 %) and pellets (28 %), usually pelleted feed produced for reindeer. Combinations of different feeds were also used during the winter, with pellets and lichens being the most frequent (27 %).

### Predators

Of all the respondents, 49 (82 %) regarded predators as the most important factor related to loss of animals, with wolverine (*Gulo gulo*; 32 %) and lynx (*Lynx lynx*; 25 %) being the two most important species, followed by eagle (species not specified but assumed to be Golden eagle, *Aquila chrysaetos*; 20 %) and brown bear (*Ursus* *arctos*; 16.6 %), as well as red fox (*Vulpes vulpes*), wolf (*Canis lupus*) and dog (*Canis familiaris*) (each of them 2.4 %).

### Other diseases than IKC

When asked about the occurrence of diseases in general, 17 % of the respondents answered that they considered infestation of the reindeer warble fly larvae (*Hypoderma tarandi*) and reindeer throat bot fly larvae (*Cephenemyia trompe*) as the most common diseases. Sixty percent of the owners conducted annual anti-parasitic treatment, whereas 19 % did not give any parasitic treatment. The treatment was usually done in September–November, and most animals in the herd were treated (both sexes, all ages). Lameness and other problems with the legs is given as the second most important cause of disease (8 %), whereas other diseases mentioned specifically in the questionnaire, such as abortion, emaciation, other parasite infestations or trauma, were not, from the herders point of view, regarded as common disease conditions.

Among the herders that had no or only restricted access to a veterinarian, 31 % responded that the parasite burden represented the most frequent disease condition, whereas those who had access to veterinary services did not to the same extent experience the parasite burden as important (P = 0.003).

## Discussion

This survey aimed at gathering traditional knowledge and experience from reindeer herders regarding IKC in reindeer, to identify herding conditions and management factors that might be associated to IKC. Due to a relatively low response rate, the results are presented predominantly in a descriptive way.

IKC in reindeer was mainly detected when animals were gathered and handled (89 %), and the main seasonal appearance of the disease was September to November. This information may be biased, since observation of disease in animals that most of the year are free-ranging in remote mountain pastures, is difficult, unless they are gathered and can be checked at close distance, and the most common season for this is during fall. Also, through herding, gathering, transport and handling, animals are exposed to stress, which may affect the immune response negatively and make them more susceptible to infections and disease [21]. This is relevant, since it has been demonstrated that experimentally immune-compromised reindeer have reactivated latent herpesvirus (CvHV2) infections [22], which has been identified as the cause of an IKC outbreak [10]. Thus, the fact that most IKC cases are reported to appear in September to November may reflect that this is when most of the animals are inspected at close range. However, it may also be a result of the disease being initiated during this period, due to the handling and the stress they experience, or a combination of the two.

Another interesting finding was that some herders claimed they had no access to a veterinarian, and that only four of 57 respondents (7 %) were considering consulting a veterinarian should disease symptoms of IKC occur. This may reflect scarce availability of a veterinarian in that area. It may also indicate a restricted willingness to spend resources on disease treatment or limited trust in the probability that veterinary advice and treatment will change the course and outcome of the disease, since most of the herders responded to IKC by slaughtering the animal. Since IKC is a transmissible disease, sometimes affecting tens or hundreds of animals, it was somewhat surprising that none of the respondents mentioned separation of the diseased animals from the healthy as a measure to restrict disease spreading.

The treatment of IKC in a reindeer herd will depend on a range of factors, such as the severity of the disease, the number of affected individuals and the herder´s ability and willingness to invest time and money on treatment and care. For some animals, displaying severe and late stage symptoms of IKC (Fig. 1d), euthanasia for animal welfare reasons may be the best solution, which 12 % of the respondents (herders) answered. Since eye ointment with antibiotics was reported as the most common treatment by the consulted veterinarian, it is of major concern that the most common and most available antibiotic drugs for eye infections in Norway are not licensed for use in production animals, including reindeer, due to lack of data on maximum residue limits (MRL). It is, however, possible to use off-label drugs to some extent, but with an extended withdrawal period.

The fact that most herders that had no or only restricted access to veterinary services also experienced the parasite burden as more significant, may indicate that they did not treat their animals against parasite infections. This may also suggest that they lack knowledge or do not receive advice on the right drugs, dose and/or timing, to optimize the effect. However, it is challenging to evaluate this, since parasites such as the warble fly larvae and the throat bot larvae, which have a negative effect on health and welfare of reindeer [23], are very common and easily recorded by the herders, whereas other health challenges or diseases may be much more difficult to identify.

Almost all the respondents in both Norway and Sweden claimed that their summer and winter pastures during 2010, the year before the survey, were of good or very good quality. Although some supplementary feeding was conducted, this indicates that among these herders, of whom 43 % observed IKC in 2010, starvation and poor pasture quality did not stand out as the main characteristic determinant for this disease. It is also important to note that herd size alone was not significantly associated to the occurrence of IKC. Most herders regarded precipitation as rain as having a negative impact on reindeer herding, during both summer and winter. This is relevant, since the effects of climatic changes in arctic and sub-arctic regions is predicted to consist of increased precipitation in the form of rain, especially associated to an increased frequency of freeze–thaw cycles during winter [18], which may hinder the animals to find their necessary food resources.

The questionnaire revealed that 21 % of the herders fed their animals regularly during the winter, whereas 64 % fed them only occasionally. Supplementary feeding has been described by herders as an emergency solution only, which may be necessary during the winter due to challenging winter pasture conditions [24]. Supplementary feeding of reindeer is costly and results in a heavier workload with more herd supervision and feed management, compared to free grazing. It may also induce a different fat composition in the meat and a taste different from the characteristic flavours of reindeer meat [24]. Further, corralling and supplementary feeding may facilitate the transmission of infectious agents among the animals [20] and also lead to feeding related disorders like diarrhoea and ruminal acidosis [25]. However, Josefsen and colleagues have found, by conducting routine reindeer necropsies over a period of time (1998–2011) in Norway, that lack of feed, hunger and emaciation was a more common cause of mortality compared to feeding related disorders [26]. Supplementary feeding may also affect the behaviour of the animals and their use of pasture, making the reindeer more dependent on human care [27]. However, it has been shown that supplementary feeding generally increases body weight and reproduction success of the females and contributes to a decrease in calf mortality [28, 29].

Somewhat surprisingly, almost 30 % of the herds represented in the survey were in contact with as many as 25 other herds or more during a year of herding (summer and winter pastures). This contact also includes exposure to circulating and enzootic infectious agents of other herds. This is in line with serological studies covering many different herding districts and regions, showing that the prevalence of some virus infections, such as pestivirus and alphaherpesvirus, has been quite stable over time [16, 30, 31]. It also suggests that managing infectious diseases should be considered a communal responsibility among herders within a district. Even if no significant association was found between the occurrence of IKC and the number of contact herds for each particular herd, the herds where IKC was reported did have a larger number of contact herds (>25). This is in line with the fact that IKC is infectious and spreads between animals and herds, although it also might reflect that the chance of having an animal with IKC probably increases with the number of animals.

In our data, we found no association between the appearance of IKC and the different methods of herding and translocation of animals between pastures, such as walking with the animals, the use of motorized vehicles on the ground, transporting them by trucks, or use of helicopter in the field, although these methods may represent different stress exposure to the animals. Also predators represent stress for free ranging animals, but in our data we did not find an association between appearance of IKC and the presence and types of predators of importance. This may reflect that there are no such associations, or that our study, due to the restricted number of respondents, lacked the power to reveal them.

## Conclusions

From this survey, it can be concluded that IKC is to be considered a common disease in reindeer (55 % of the herds in 2010), typically affecting 1–5 animals at a time, and appearing most often during September to November. The chance of having registered the disease in a herd was higher for herds having >25 contact herds. In spite of the contagious nature of IKC, none of the herders responded that they isolated affected animals from healthy, and the majority of the herders usually slaughtered affected animals rather than consulting a veterinarian for medical treatment. The herder´s experience, that precipitation as rain had a negative impact on reindeer herding, is of relevance when investigating possible effects of climatic changes in arctic and sub-arctic regions. We recommend herders to be aware of this disease, recognize the initial symptoms, isolate affected animals from healthy, and to make use of local veterinary expertise in order to safeguard animal welfare and limit the spread and economical loss due to having a disease outbreak in the herd.
